# HTS-Based Diagnostics of Sugarcane Viruses: Seasonal Variation and Its Implications for Accurate Detection

**DOI:** 10.3390/v13081627

**Published:** 2021-08-17

**Authors:** Martha Malapi-Wight, Bishwo Adhikari, Jing Zhou, Leticia Hendrickson, Clarissa J. Maroon-Lango, Clint McFarland, Joseph A. Foster, Oscar P. Hurtado-Gonzales

**Affiliations:** 1 USDA-APHIS Plant Germplasm Quarantine Program, Beltsville, MD 20705, USA; bishwo.n.adhikari@usda.gov (B.A.); brynleti@gmail.com (L.H.); joseph.a.foster@usda.gov (J.A.F.); 2 Department of Agriculture, Agribusiness, Environmental Sciences, Texas A&M University-Kingsville, Kingsville, TX 78363, USA; jing.zhou@tamuk.edu; 3 USDA-APHIS-PPQ-Emergency and Domestic Program, Riverdale, MD 20737, USA; clarissa.j.maroon-lango@usda.gov; 4 USDA-APHIS-PPQ-Field Operations, Raleigh, NC 27606, USA; clint.d.mcfarland@usda.gov

**Keywords:** sugarcane virus, quarantine, seasonal variation, sequencing depth, metagenomics

## Abstract

Rapid global germplasm trade has increased concern about the spread of plant pathogens and pests across borders that could become established, affecting agriculture and environment systems. Viral pathogens are of particular concern due to their difficulty to control once established. A comprehensive diagnostic platform that accurately detects both known and unknown virus species, as well as unreported variants, is playing a pivotal role across plant germplasm quarantine programs. Here we propose the addition of high-throughput sequencing (HTS) from total RNA to the routine quarantine diagnostic workflow of sugarcane viruses. We evaluated the impact of sequencing depth needed for the HTS-based identification of seven regulated sugarcane RNA/DNA viruses across two different growing seasons (spring and fall). Our HTS analysis revealed that viral normalized read counts (RPKM) was up to 23-times higher in spring than in the fall season for six out of the seven viruses. Random read subsampling analyses suggested that the minimum number of reads required for reliable detection of RNA viruses was 0.5 million, with a viral genome coverage of at least 92%. Using an HTS-based total RNA metagenomics approach, we identified all targeted viruses independent of the time of the year, highlighting that higher sequencing depth is needed for the identification of DNA viruses.

## 1. Introduction

Plant pathogens play a significant role in affecting the yield and the quality of agricultural products. A recent worldwide survey on five major food crops (wheat, rice, maize, potato, and soybean) found that 137 plant pathogens and pests caused an estimated yield loss ranging from 17 to 30% of crop productivity globally [1,2]. The annual economic losses caused by virus infections alone were estimated at over 30 billion dollars worldwide [3,4]. Globalization has led to the increasing intercontinental trade of agricultural products and the expanded utilization of imported germplasm for breeding purposes over the last few decades [5,6]. The widespread distribution of plant pathogens has been attributed to long-distance movement of plant material across borders, often resulting in the establishment of plant pathogens in new territories followed by significant economic losses [2]. One striking example is the plum pox virus, a Potyvirus first reported in Bulgaria in the early 1930s that caused the disease known as “Sharka” in Prunus. Since then, this devastating virus has spread to major stone fruit production areas around the world, including the U.S. It took the U.S. over 53 million dollars and twenty years of effort to finally eradicate the virus by 2019 [7,8,9,10]. It is therefore essential to implement international and national-level strategies that can prevent and mitigate the spread of foreign pathogens [11]. A critical role of these strategies is to enable the release of pathogen-free germplasm and ensure their safe incorporation into breeding programs and agricultural systems while minimizing potential adverse economic and trade impacts [1,12].

The Plant Germplasm Quarantine Program (PGQP) of the U.S. Department of Agriculture-Animal and Plant Health Inspection Service (USDA-APHIS) is the largest federal plant quarantine center in the U.S. and serves as the portal for the legal and safe release of prohibited plant materials imported into the country. The USDA-APHIS regulates the importation of prohibited plant genera under the Plant Quarantine Act of 1912, The Organic Act of 1944, The Federal Plant Pest Act of 1957, and the Federal Noxious Weed Act of 1974. The PGQP imports these plants and tests them for the presence of regulated bacteria, viruses, viroids, and phytoplasmas through four major programs: the Poaceae, Pomes and Prunus, Vegetables, and Woody Ornamentals quarantine programs. These programs process more than 20 economically important plant genera including Ipomoea, Malus, Manihot, Miscanthus, Oryza, Prunus, Pyrus, Saccaharum, and Solanum [13]. The Poaceae quarantine program manages the importation and processing of bamboos, forage grass, ornamental grass, rice, sorghum, and sugarcane germplasm. Among these regulated plant species, sugarcane has been cultivated in over 100 countries worldwide due to its value for high sucrose content and bioenergy use [14,15]. Sugarcane, however, is heavily infected by several viruses that severely compromise its production, hence threatening the sugarcane-based industries. The virus-triggered economic losses on sugarcane result, in part, from its vegetative propagation via stalk cuttings, which favors the transmission of viral pathogens in nurseries and commercial fields [16,17].

Current diagnostic workflows include conventional indexing using indicator plants, serological tests (ELISAs), PCR-based techniques, and electron microscopy. These techniques are well established and are utilized by plant quarantine and disease diagnostic programs throughout the world. For many years, these assays have been successfully implemented and validated to identify an array of well-characterized quarantine pathogens within the context of quarantine standards because of their specificity to target pathogens and the ability to detect systemic pathogens. However, these conventional techniques require a priori knowledge of pathogens for accurate detection and lack flexibility to detect emerging genetic variants and mixed infections. These limitations make the conventional techniques less adaptable to the detection of pathogens on a growing number and type of prohibited plants requiring quarantine. This challenge underscores the necessity of developing effective broad-spectrum detection methods that could be employed to detect an expandable list of quarantine plant pathogens. On the other hand, the advent of high-throughput sequencing technologies (HTS) have revolutionized the identification and characterization of plant pathogens over the last decade [18,19]. Since its debut in identifying novel viruses infecting grapevine and several other plant species, an increasing number of plant viruses and viroids have been fully or partially characterized using HTS-based techniques, greatly improving virus detection, disease diagnosis, and the study of diseases with unknown etiology [18,20,21,22,23,24,25,26]. There are several advantages of HTS when compared with conventional detection methods, including its capability to identify both anticipated and unknown pathogens without acquiring prior knowledge of their presence in the specimen [19,27], its reduced timeframe, and enhanced comprehensiveness in virus detection by a direct comparison with biological indexing [28]. The comprehensiveness of HTS outperforms other conventional detection methods given that, in a single test, it can capture the sequences of all viruses present, including the co-existence of multiple viral species or strains of the same species. Despite the successful application of HTS in the discovery and characterization of novel viruses, the establishment of HTS-based routine diagnostics platform for plant viruses is still evolving, facing both biological and technical challenges [29,30]. The decreasing cost of sequencing has made HTS a more widely used technique for virus detection, a reliable way to validate conventional assays, and an exploratory tool to detect novel viruses [31]. However, limited data is available in terms of the experimental performance of HTS in routine testing [32], which is a critical component in the evaluation of its implementation as a routine and accurate diagnostic tool for its use in quarantine programs.

Seasonal fluctuations of virus accumulation in plants is likely to happen given that viral replication and plant growth are highly temperature-dependent [33]. Studies have suggested that seasonal changes could be another important factor contributing to virus-host interactions [34]. Although the role of antiviral mechanisms, such as RNA silencing and systemic acquired resistance in the seasonal virus variations, remains elusive [33], studies have revealed diminished viral titers and disease symptoms at elevated temperatures (>25 °C). Such higher temperature-induced enhancement in RNAi-mediated antiviral resistance [35,36,37] suggest that summer is not an optimal time for sensitive virus detection. On the other hand, it is also known that a temperature below 15 °C is not optimal for replication of plant viruses either [38,39,40,41]. Taking this into consideration, diagnostic testing and indexing of quarantine held Poaceae samples was conducted in the spring and the fall for two consecutive years. In this study, we evaluated the use of HTS as a diagnostic tool for the virus detection of seven sugarcane viruses of regulatory relevance for the U.S. sugarcane industry. We applied similar parameters under which conventional diagnostics tests, such as PCR-based tests, are validated in the PGQP. This validation included comparing the seasonal sampling effect (spring and fall) while adding the sequencing depth needed for the detection on seven sugarcane viruses. We report on the robustness of the metagenomic sequencing of ribo-depleted HTS libraries for the detection of both RNA and DNA sugarcane viruses and determine that the spring season is the optimal time for reliable diagnosis of all tested quarantine sugarcane viruses. Additionally, we discuss the impact of sequencing depth in the detection of different types of viruses. A better understanding of the seasonal virus accumulation pattern and the incorporation of HTS analysis into existing biological indexing methods will improve the current quarantine framework for improved pathogen detection. 

## 2. Materials and Methods

### 2.1. Plant Materials and Seasonal Sample Collection

Nine sugarcane samples (*Saccharum* spp., dubbed P1 through P9) infected with sugarcane striate mosaic associated virus (SCSMaV), sugarcane yellow leaf virus (SCYLV), sugarcane mosaic virus (SCMV), sugarcane streak mosaic virus (SCSMV), fiji disease virus (FDV), sugarcane streak Egypt virus (SCSEV), and sugarcane white streak virus (SCWSV) were used in this study (Table 1). Plants were maintained in greenhouses at the USDA-APHIS Plant Germplasm Quarantine at Beltsville, Maryland, and have been routinely used as positive virus controls for conventional indexing and bioassays. The selected samples belong to the Poaceae positive control collection of sugarcane germplasm and originated from different geographical regions including Africa, Asia, North America, and Australia/Oceania. Leaf tissue from each plant was harvested twice, appropriately six months apart, during the spring (March–May) and fall (September–November) seasons of 2017 and 2018. Regardless of virus symptoms, each plant was sampled by collecting three leaves, ranging from the fully matured to the newly emerged ones. The presence of the studied viruses in individual plant was confirmed by PCR or RT-PCR prior to sequencing.

### 2.2. RNA Extraction, Library Preparation and Sequencing

Total RNA was extracted as previously reported [42] using the RNeasy Plant Mini Kit (Qiagen, Germantown, MD, USA). Extracted RNA was quantified using the Qubit 3.0 fluorometer (Life Technologies, Carlsbad, CA, USA) and quality assessed using the Agilent 4200 TapeStation system, RNA ScreenTape assay (Agilent Technologies, Santa Clara, CA, USA). A minimum RNA integrity number (RIN) of 6 was applied as the cutoff prior to HTS library construction. Eighteen single-indexed cDNA libraries corresponding to nine plant specimens, each with samples prepared from the spring and fall seasons, were constructed from ribosomal RNA-depleted extracts synthesized using the TruSeq Stranded Total RNA with Ribo-Zero Plant Kit (Illumina, San Diego, CA, USA), according to the manufacturer’s instructions. Library quantification and quality assessment were performed using the Qubit 3.0 fluorometer (Life Technologies, Carlsbad, CA, USA) and Agilent 4200 TapeStation system, High Sensitivity D1000 ScreenTape assay (Agilent Technologies, Santa Clara, CA, USA), respectively. After quantification and normalization, the 18 libraries were independently sequenced on an Illumina NextSeq 500 instrument using a 75-cycle high output sequencing cartridge (Illumina, San Diego, CA, USA).

### 2.3. HTS Data Analysis: Normalization, Mapping, and De Novo Assembly

Raw data files in binary base call (BCL) format were first converted to FASTQ format; the data was then demultiplexed by removing adaptors and barcodes using Illumina bcl2fastq2 V2.20 (Illumina, San Diego, CA, USA), allowing zero barcode mismatches. Reads were prepared for downstream analyses by importing FASTQ files into CLC Genomics Workbench Version 11.0.1 (CLC Bio, Qiagen, Germantown, MD, USA) and trimmed using the following parameters: Trim quality score limit = 0.05; Trim ambiguous nucleotides with maximum number of ambiguities = 2. Trimmed reads were de novo assembled using CLC Genomics Workbench with the following parameters: Word size = 20; Bubble size = 50; Minimum contig length = 75. Resultant contigs were compared to a custom-built viral genomic (BLASTn) and proteomic (BLASTx) databases using the BLAST (Basic Local Alignment Search Tool) algorithm in CLC Genomics Workbench with the following parameters: Expect = 10; Match = 2; Gap costs = Existence 5; Extension = 2; Filter low complexity = yes; Word size = 11; Mismatch = −3; Max number of hit sequences = 3. For each sample, the top viral hit genome with E-value = 0 was extracted from the BLAST results. The BLAST results were confirmed by mapping the trimmed reads against the extracted reference genome of the corresponding top viral genome originated from NCBI accession. Using CLC Genomics Workbench, mapping was conducted with the following parameters: Mismatch cost = 2; Insertion cost = 3; Deletion cost = 3; Length fraction = 0.5; Similarity fraction = 0.80. Mapping tracks, detailed mapping reports, and summary mapping reports were generated using NGS Core Tools in CLC Genomics Workbench. The number of reads mapped to the reference genome, percentage of the reference genome covered, reference genome size, and average genome coverage was used to identify false positive results. To further corroborate the identification of the possible viral hits, a consensus sequence was extracted from each viral pathogen mapping track, and a similarity search was performed against NCBI non-redundant databases with BLASTn and BLASTp (NCBI BLAST). When the viral consensus sequence showed significant similarity to a known viral genome (BLASTn E-value = 0) or contained a putative conserved domain as predicted by BLASTp, the result was considered a positive viral hit. Any viral hit with E-values above zero and non-significant match to putative conserved domains was considered as a possible false positive. Open reading frames of the consensus were identified using Classical Sequence Analysis in CLC Genomics Workbench.

### 2.4. Normalized Read Count Calculation and Read Sub-Sampling

In order to correct for differences associated with sequencing depth and the length of viral genome, normalized read counts were calculated as reads per kilobase of the reference sequence per million reads (RPKM) values by taking into consideration (a) the length of the viral reference genome, (b) the number of reads mapped to each genome, and (c) the total number of reads per sample [43]. In this analysis, RPKM value was calculated using the following formula: number of reads mapped to a genome/(length of the genome/1000 × total number of reads/1,000,000). RPKM values from the spring and fall seasons for each plant specimen, interpreted as viral read abundance per sample [44,45,46], were compared. No threshold regarding RPKM value was applied.

To estimate the minimum number of reads required to detect an individual virus in two different seasons, sequence reads from each accession were randomly sub-sampled using the sampling tool in CLC Genomics Workbench. Reads were sub-sampled at different levels ranging from 0.5 to 25 million reads. Sub-sampling was repeated three times per sample/size combination, yielding a total of 270 datasets (9 plant accessions × 5 sub-sample sizes × 2 seasons × 3 replicates of sub-sampling). For each sub-sample, the CLC Genomics Workbench de novo assembly, mapping, and validation pipeline described above was used to identify the viral pathogens. The minimum number of reads required to detect a viral pathogen in any season was determined using the lowest read sub-sample, in which mapped reads covered 60–80% (DNA viruses) or greater than 80% (RNA viruses) of the viral pathogen reference genome.

### 2.5. Quantitative RT-PCR of Targeted Viruses

In order to validate the seasonal viral titer variation revealed by HTS, Taqman^®^ RT-qPCR assays were designed for individual viruses by targeting their respective coat protein/capsid protein genes (Table S2). For each virus, multiplex RT-qPCR was performed by incorporating an internal control (Table S2, courtesy of USDA APHIS S&T Beltsville Laboratory) and using SuperScript III Platinum One-Step RT-qPCR kit (ThermoFisher, Waltham, MA, USA) according to the manual. The 20 µL reaction has the final concentration of virus-specific forward primer: reverse primer: probe at 0.4 µM: 0.4 µM: 0.2 µM and internal control forward primer: reverse primer: probe at 0.3 µM: 0.3 µM: 0.15 µM, respectively. Two technical replicates were included for each sample when conducting RT-qPCRs. The program was initiated at 50 °C for 15 min then at 95 °C for 2 min, followed by 40 cycles each consisted of 95 °C for 10 s and 55–58 °C for 30 s. Relative quantification was calculated according to Livak and Schmittgen [47] using Design and Analysis Software version 2.4 (ABI, Thermofisher, Carlsbad, CA, USA).

## 3. Results

### 3.1. Identification of DNA and RNA Viruses: Comparison between HTS and Conventional Indexing Methods

In order to determine whether HTS could consistently detect regulated sugarcane viruses by the USDA-APHIS, we selected nine plant specimens kept as positive controls and sequenced them in spring (March–May) and fall (September–November) during the years 2017 and 2018. HTS identified DNA and RNA viruses in both seasons (Table 1) in consistency with the conventional indexing methods. Two out of the nine plant samples were identified as co-infected with at least two viruses, whereas the rest were confirmed as single-virus infection (Table 2). Furthermore, the HTS methodology employed in this study could identify viruses with diverse genomic organizations including positive, single-stranded RNA, double-stranded RNA, and single-stranded DNA.

The total number of reads obtained for each sample varied between 24.5 and 42.7 million, which was sufficient to achieve viral genome coverage greater than 99% for all but SCWSV, where genome coverage for spring and fall was 94% and 79%, respectively (Table S1). The percentage of viral reads identified in individual sample was significantly higher for RNA viruses (ranging from 0.04% to 5.37%) compared with DNA viruses (ranging from 1.30 × 10^−3^% to 0.03%) (Table 3).

### 3.2. Comparison of Viral RPKM-Normalized Reads between Seasons

A critical component in the use of HTS as a diagnostic tool is to determine the optimum season to perform HTS during which the viral titer is higher. For this reason, RPKM-normalized viral reads, which serves as a semi-quantitative measurement of viral titer due to its high correlation with virus load within samples [44,45,46], were compared between the spring and fall seasons.

#### 3.2.1. RNA Viruses

**Figure 1:**
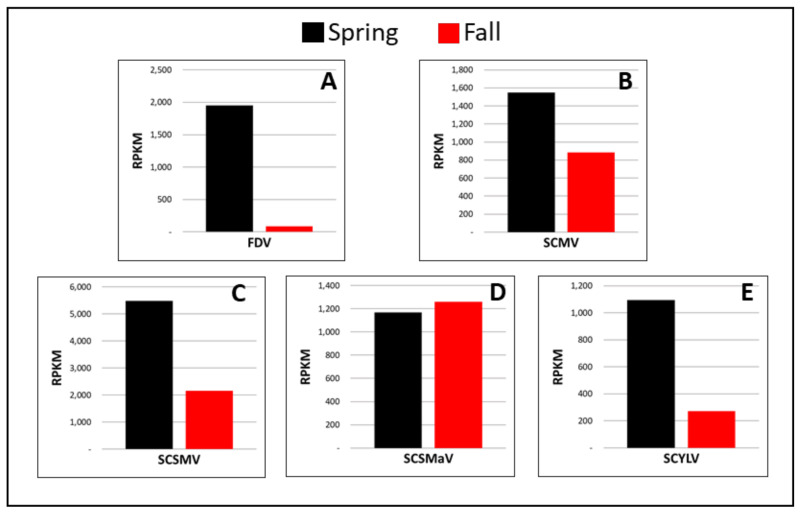
Spring and fall viral read comparison of six RNA viruses in sugarcane quarantine specimens. Viral reads are normalized as RPKM, reads per kilobase per million, to account for viral genome size [43]: (**A**) Fiji disease virus (FDV); (**B**) sugarcane mosaic virus (SCMV); (**C**) sugarcane streak mosaic virus (SCSMV); (**D**) sugarcane striate mosaic associated virus (SCSMaV); (**E**) sugarcane yellow leaf virus (SCYLV), represented by sugarcane sample P8.

Fiji disease virus (+dsRNA, family Reoviridae). Sugarcane sample P4 was imported from Australia as a positive control for FDV. The FDV genome coverage was 100% in both seasons (Figure 1A; Table 3). Among all viruses identified by HTS in this study, FDV showed the largest seasonal variation in terms of viral titer where viral read percentage in spring was 0.89% as compared to 0.04% in the fall, representing a 23.25-fold difference (Figure 1A; Table 3). 

**Figure 2:**
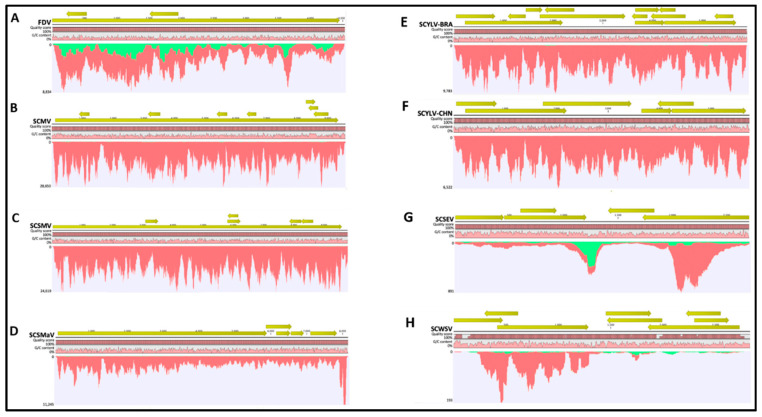
Genome coverage of RNA (**A**–**F**) and DNA (**G**,**H**) viruses from sugarcane specimens sampled in the spring. Sequencing reads from RNA and DNA viruses were mapped to best hit reference genomes using CLC Genomics Workbench at default parameters. Shown in each panel are open reading frames, consensus sequence, sequence quality score, G/C content, and coverage graph. The SCYLV-BRA genotype is represented by sample P1. The SCYLV-CHN genotype is represented by sample P6. Colors in the map denote the single reads mapped in their forward (green) and reverse (red) directions. ORFs are denoted by greenish yellow arrows.

Sugarcane mosaic virus (+ssRNA, family Potyviridae). SCMV was originally identified in a sugarcane specimen imported from South Africa (P1). P1 was found to be co-infected with SCYLV (Table 2 and Table 3) based on HTS. SCMV genome coverage was 100% in both seasons and 96% identical to the reference sequence (Figure 2B; Table 3); the percentage of viral reads in spring was higher (4.31%) than in the fall (2.46%), accounting for a 1.8-fold titer increase in spring (Figure 1B; Table 3). 

Sugarcane streak mosaic virus (+ssRNA, family Potyviridae). Sugarcane sample P7, imported from Pakistan, was infected with SCSMV. Consistent with SCMV, which also belongs to the Family Potyviridae, the RPKM values of SCSMV were higher in spring than in fall, with a 2.55-fold increase while the genome coverage reached 100% in both seasons (Figure 1C and Figure 2C; Table 3).

Sugarcane striate mosaic associated virus (+ssRNA, family Betaflexiviridae). SCSMaV was present in sugarcane sample P3, imported from Australia. Genomic coverage of SCSMaV was 100% in both seasons (Figure 2D; Table 3). Viral reads accounted for 0.95% and 1.03% of the total number of reads obtained in spring and fall, respectively (Table 3), suggesting that the viral titer remained almost the same between the two tested seasons, with a slightly higher level (7.9% increase) in the fall season (Figure 1D). 

Sugarcane yellow leaf virus (+ssRNA, family Luteoviridae). SCYLV was identified in sugarcane samples P1, P2, P6, P8, and P9 (Table 2). Sample P1, imported from South Africa, was co-infected with SCMV. Samples P2, P6, P8, and P9 were single-infected with SCYLV and originated from Papua New Guinea, the U.S., and Guatemala (P8 and P9), respectively. The genome coverage for all four SCYLV isolates was between 98–100% (Figure 2E,F; Table 3) and the ratio of RPKM values in spring to that in the fall season varied between 3.58 and 16.80 (Figure 1E and Figure 4), suggesting virus accumulation at a much higher level in the spring season regardless of the SCYLV genotypes.

#### 3.2.2. DNA Viruses

**Figure 3:**
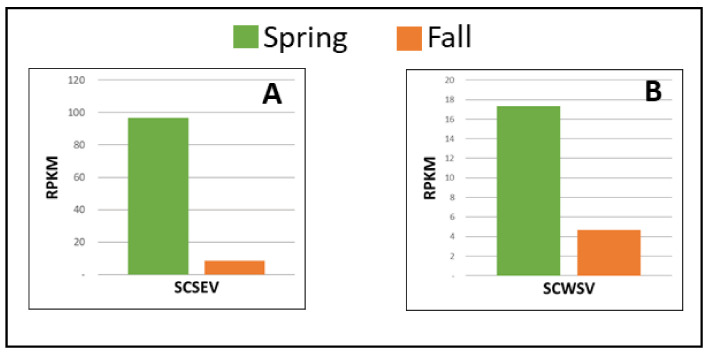
Spring and fall viral read comparison of two DNA viruses in sugarcane quarantine specimens. Viral reads are normalized as RPKM, reads per kilobase per million, to account for viral genome size [43]: (**A**) sugarcane streak Egypt virus (SCSEV); (**B**) sugarcane white streak virus (SCWSV).

Sugarcane streak Egypt virus and sugarcane white streak virus (ssDNA, family Geminiviridae). Sugarcane sample P5 was co-infected with SCSEV and SCWSV. Similar to SCYLV, SCMV, SCSMV, and FDV, the percentage of SCSEV HTS-derived viral reads identified in spring was higher than in fall, accounting for a 10.8-fold RPKM increase (Figure 3A; Table 3). SCSEV genome coverage was 100% in both seasons, whereas the genome coverage for SCWSV was 94% and 79% in the spring and fall seasons, respectively. Similar to the seasonal changing pattern of SCSEV, the percentage of SCWSV reads identified in spring was higher than in fall, accounting for a 3.4-fold viral titer increase (Figure 3B, Table 3).

### 3.3. Validation of Seasonal Viral Titer Change Using RT-qPCR

To validate the seasonal fluctuation of viral titer detected by HTS, RT-qPCR assays targeting the coat protein/capsid protein genes were designed. Quantification of the expression level of virus-specific genes and comparing that between the spring and fall seasons in individual samples revealed higher viral titer in the spring than in the fall season for all viruses except for SCSMaV, which showed a small titer increment (8.8%) in the fall. This trend agrees with HTS analysis, though with a wider fold change ranging from 1.20 to 77.9 for the six viruses (SCYLV, SCMV, SCSMV, FDV, SCWSV, and SCSEV). In addition, RT-qPCR also revealed FDV as the virus having the biggest seasonal variation. 

### 3.4. Reproducibility: Comparison of SCYLV RPKM Variation

**Figure 4:**
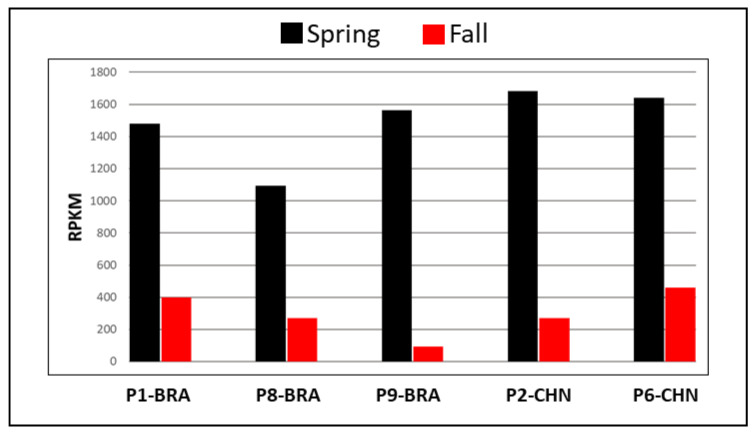
Spring and fall viral read comparison of two sugarcane yellow leaf virus (SCYLV) genotypes, Brazil (BRA) and China (CHN), in five sugarcane quarantine specimens. Viral reads are normalized as RPKM, reads per kilobase per million, to account for viral genome size [43]. The SCYLV-BRA genotype is represented by three samples (P1, P8, and P9), and the SCYLV-CHN genotype is represented by two samples (P2 and P6).

To assess the reproducibility of the RPKM variation observed in this study, we selected five single- or co-infected SCYLV specimens. These samples were imported from diverse geographical locations, allowing us to determine if this variation is independent of germplasm origin or viral genotype (Table 2). HTS analyses showed that samples P1, P8, and P9 were infected with SCYLV-BRA genotype, while samples P2 and P6 were infected with SCYLV-CHN genotype (Table 2 and Table 3). The genomic coverage for all five SCYLV isolates were above 99%, independent of the season during which the analysis was performed. RPKM values obtained from all five samples were higher in spring when compared to the fall season (Figure 4), with a fold increase between 3.58 and 16.80. The seasonal variation in viral titer was independent of viral genotype, germplasm origin, or whether the sample was single-infected (samples P8 and P9) or co-infected with SCMV (samples P1, P2, P6). Similar analysis for other viruses was not possible due to the absence of multiple sugarcane accessions carrying the other six viruses.

### 3.5. Seasonal Viral Identification: Comparison of Sequencing Depth

**Figure 5:**
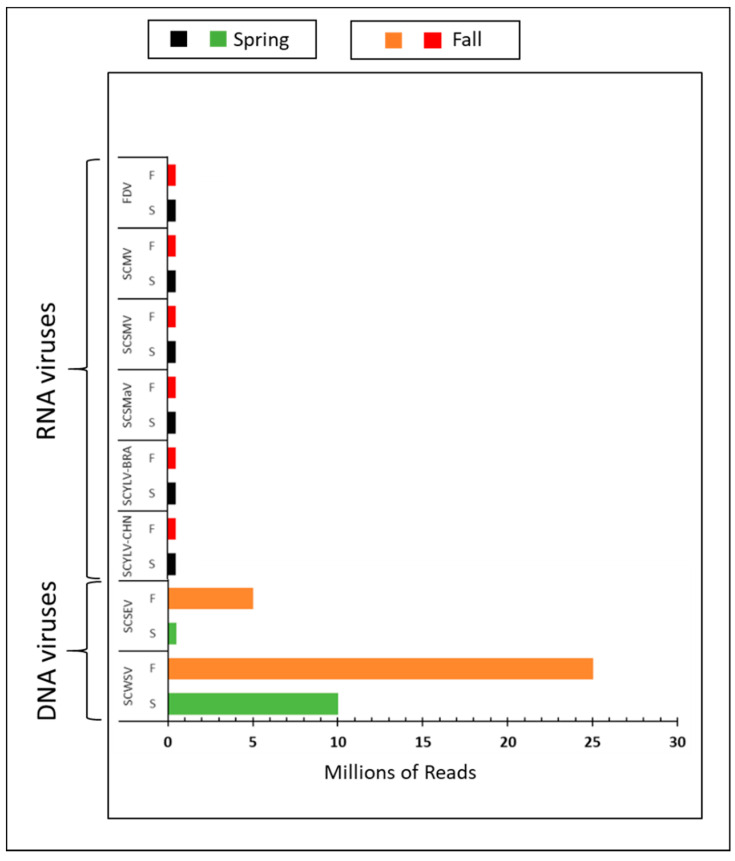
Sequencing depth required for RNA and DNA virus identification in spring and fall. Viral pathogens were identified using CLC Genomics Workbench to analyze randomly sub-sampled sets of 0.5, 1, 5, 10, 20, and 25 million reads. The limit of detection was defined as the smallest sub-sampled set in which mapped reads covered 60–80% (DNA viruses) or ≥80% (RNA viruses) of the best hit viral genome. The SCYLV-BRA genotype is represented by sample P1. The SCYLV-CHN genotype is represented by sample P2.

In order to determine the number of reads sufficient to detect individual target viruses, we randomly sub-sampled three replicates of five sub-sample sizes (0.5, 1, 5, 10, 20 million reads) from the total number of reads per each of the nine samples. Based on this analysis, we determined that the minimum number of reads required for virus detection during fall and spring ranged from 0.5 to over 20 million (Figure 5). A trend was observed across tested viruses: RNA viruses can be detected below 0.5 million reads, whereas for DNA viruses, the minimum number of reads required for detection was much higher, except for the SCSEV spring specimen. For all of the RNA viruses tested in this study (SCSMaV, SCYLV-BRA, SCYLV-CHN, SCMV, SCSMV, and FDV), as few as 0.5 million reads was sufficient to achieve over 92% genome coverage in both the fall and the spring seasons. On the other hand, for SCSEV, one of the DNA viruses, there was an obvious lack of high variability in the number of reads required to detect SCSEV in different seasons: 0.5 million reads was sufficient to achieve 84% genome coverage in the spring, whereas 1 million reads could only cover 46% of genomes during the fall. An even higher number of reads was needed to detect SCWSV, another DNA virus: 10 million reads was required to cover 87% of the genome in the spring, whereas only 73% of the genome could be covered in the fall. Due to the low coverage in the fall, another read sub-sample bin was added by randomly sub-sampling 25 million reads from the total reads. Despite high read numbers (25 million reads), the genome coverage remained lower than 80% for SCWSV. Based on this empirical evidence, the genome coverage threshold for RNA and DNA viruses were determined as being above 80% and 60–80%, respectively.

## 4. Discussion

The validation of HTS as a reliable virus diagnostic tool of different sugarcane plant viruses of quarantine relevance was assessed using sugarcane specimens sampled during the spring and fall seasons. The total number of reads obtained under a ribo-depleted approach varied between samples, as did the abundance of viral reads per sample, indicated by RPKM values (Table 3). The viral reads obtained for each sample was sufficient to achieve near-complete coverage of viral genomes (over 99%) for all viruses except SCWSV.

We chose to use the rRNA-depleted total RNA approach in this study because of the longer contigs and higher genome coverage it generates [48,49]. When comparing total RNA and siRNA approaches, a higher proportion of viral genomes covered by de novo generated contigs have been reported from total RNA [48,50]. The lower coverage of viral genomes by siRNA-derived de novo contigs is likely due to the short contig length cutoff, which should stay under 60 nt in order to avoid eliminating shorter contig reads and thus improving the sensitivity of the approach [51]. As a result, short read length increases the possibility of producing false positive results and incorrect assembly [52]. 

Another layer of complexity of the siRNA approach is that the generation of siRNA is a host- and virus-specific process. The degrees of virus infection and host response to infection are most likely to affect viruses present in the siRNA dataset, contributing towards the variation of genome coverage between viruses [48,52]. On the contrary, the total RNA approach outperforms its siRNA HTS-virus detection in terms of identification of co-infection by multiple virus species or virus strains of the same species in a single plant sample, a situation that is not uncommon [51,52]. In our study, all seven viruses detected by HTS, sampled in different seasons, were confirmed by PCR (Table S3). This suggests that the HTS-total RNA shotgun metagenomics can consistently identify the same viruses as the conventional testing methods used during virus indexing of sugarcane samples, regardless of the virus genomic material. The fact that HTS showed higher fold changes in viral titers between the fall and spring seasons indicates a higher sensitivity of this method over conventional methods. 

Seasonal variations of virus abundance have been well documented in animal- and human-infecting viruses as a critical component of viral epidemiology [53,54,55,56]. In contrast, very little is known about plant virus dynamics across seasons in either natural or controlled environments, despite a high significance of such information in plant virus detection and viral disease management. Nevertheless, studies have shown that higher temperatures trigger host RNA silencing mechanisms that inhibit virus accumulation on different plant species in greenhouse experiments [35,36,37,40,57], possibly suggesting that summer is not the optimum time for virus detection. With this in mind, we used the RPKM-normalized viral reads generated by HTS analysis as a proxy for viral titer to quantify the levels of seven regulated sugarcane viruses during the spring and fall seasons. This validation followed the current diagnostics workflow at the PGQP for Poaceae germplasm, which requires the indexing of imported plants during the spring and fall seasons. Six viruses, including SCYLV, SCMV, SCSMV, FDV, SCWSV, and SCSEV exhibited higher levels of viral titer in spring compared to fall. The only exception was SCSMaV, where RPKM-normalized viral reads revealed a relatively consistent level of accumulation between seasons, with a slight increase (7.9%) in the fall. Validation of these results using RT-qPCR also revealed higher levels of accumulation during the spring season for the same six viruses, as revealed by HTS. Consistent with HTS analysis, RT-qPCR results also showed a small increment (8.8%) in SCSMaV titer in the fall as compared to spring. In terms of the sequencing depth required to confidently detect target viruses, no more than 0.5 million reads were sufficient to achieve over 92% genome coverage in both the fall and the spring seasons for all RNA viruses (SCSMaV, SCYLV-BRA, SCYLV-CHN, SCMV, SCSMV, and FDV). A similar finding was reported by Visser et al. [48] after analyzing the genome coverage of two Closteroviruses by sub-sampling the total reads generated by metagenomic sequencing of ribo-depleted RNA. Their results also indicated that as few as 0.5 million reads were enough to provide complete genome coverage of both Closteroviruses [48]. In contrast to RNA viruses, the number of reads required to detect DNA viruses was much higher and less consistent between seasons: for SCSEV, 0.5 million reads were sufficient to achieve 84% genome coverage in the spring, whereas 1 million reads provided only 46% genome coverage during the fall (Table S1). An even higher number of reads was needed to detect SCWSV: 10 million reads were required to cover 87% of the genome in the spring, whereas only 73% of the genome could be covered in the fall with reads as high as 20 million (Table S1). Such a discrepancy in sequencing depth between RNA and DNA viruses has also been previously observed [50]. Randomly selected subsample reads from total RNA metagenomics sequencing were mapped to RNA- and DNA viruses, showing that 10 million reads were sufficient to cover the complete RNA viral genomes, whereas the genome coverage was less than 80%, even at the highest level of sampling (50 million reads) [50]. 

The difference in the sequencing depth required to detect RNA and DNA viruses might lie in the fact that the HTS-total RNA shotgun metagenomics method relies on assembling genome-wide RNA nucleotide reads for virus identification. However, the working targets of this method—the RNA molecules—are only present in limited abundance for DNA viruses, in the form of mRNA transcribed from viral genome [58]. In contrast, in the case of RNA viruses, both viral genomic- and complementary viral genomic-strands could function as templates during the initial stages of library preparation, thus enhancing the abundance of sequencing reads and coverage across viral reference genomes [58,59,60]. It is noteworthy that RNA viral genomes exhibit evenly distributed reference genome coverage as compared to DNA viruses, where sequencing reads were highly clustered and uneven (Figure 2). Our study also showed that the differences in sequencing depth required to detect DNA versus RNA viruses is much higher than the differences indicated by Ct values (Tables S1 and S3). For instance, it required between 5 and 10 million reads to detect SCWSV in samples collected during the fall (Table S1), indicating a 10–20-fold change higher than the sequencing depth required to detect FDV in the fall. However, the Ct values of the two samples were very similar (Table S3). 

HTS is a promising tool to screen large-scale samples for potential pathogen infections within a limited timeframe, given that it can depict the complete view of the viral phytosanitary status of a plant [30]. The comprehensiveness of this technique is critical for national plant quarantine programs such as the PGQP, where the type and number of prohibited plants requiring quarantine is consistently growing as a result of an increasing global exchange of agriculture-related products. In comparison, the fact that current quarantine procedures only test for well-characterized pathogens prevalent in the country of origin and elsewhere could leave unknown viruses and virus-like agents or emerging genetic variants undetected [23]. The current workflow processing the virus-diagnostics at the PGQP for Poaceace crops requires biological indexing, including serological tests, PCR, RT-PCR, and bioassays using indicator plants during two seasons (spring and fall) for two-consecutive years. One major consideration of using HTS as a routine detection assay is whether it is sensitive enough compared to conventional detection assays. Comparison between the results of our routine PCR-based tests and HTS data demonstrated that the same viruses were detected using both methods. This demonstrates that our HTS-based platform is a sensitive tool for detecting the sugarcane viruses presented in this study in a single test, in comparison to conventional diagnostics tests. The success of consistently detecting seasonal titer changes of two SCYLV genotypes, imported or originated from different countries and from different sugarcane genotypes, further indicated this technology could produce results with high reproducibility. 

While our results present an interesting and evolving development in the application of HTS for plant virus detection, the limitations of the study should also be acknowledged. As with other metagenomic analyses, our results are based on the analysis of five RNA and two DNA viruses from plants maintained under greenhouse conditions. Therefore, results may not always reflect what happens in the real field. Because the current study included biological replicates for ScYLV, similar analyses with replicates will be required for other viruses. Because of the low abundance of reads from DNA viruses, it is possible that a significantly higher number of total reads may be required to detect DNA viruses to reach the coverage observed for RNA viruses. Regardless of the type of virus to be detected, caution should be practiced while applying these findings to other crops. Nevertheless, the assessment of seasonal virus accumulation levels revealed in this study suggest that spring is the optimal sampling season for sugarcane viruses, both for better detection and shorter quarantine processing times. In summary, the outcome of the current study provides convincing evidence that HTS-based diagnostics can consistently identify all the targeted viral pathogens in a single test in a fashion complementary to the existing biological indexing methods.

## Figures and Tables

**Table 1:** Sugarcane RNA and DNA viruses used in this study.

Virus Species	Viral Family	Genome
Fiji disease virus (FDV)	*Reoviridae*	dsRNA
Sugarcane mosaic virus (SCMV)	*Potyviridae*	+ssRNA
Sugarcane streak mosaic virus (SCSMV)	*Potyviridae*	+ssRNA
Sugarcane striate mosaic associated virus (SCSMaV)	*Betaflexiviridae*	+ssRNA
Sugarcane yellow leaf virus (SCYLV)	*Luteoviridae*	+ssRNA
Sugarcane streak Egypt virus (SCSEV)	*Geminiviridae*	ssDNA
Sugarcane white streak virus (SCWSV)	*Geminiviridae*	ssDNA

**Table 2:** Viruses identified by HTS in sugarcane positives controls in spring and fall.

Sample	Origin	Season	Virus Identified ^1^	NCBI Accession	Viral Genome (bp)
P1	South Africa	Spring	SCYLV-BRA	AF157029	5899
			SCMV	JX237862	9571
		Fall	SCYLV-BRA	AF157029	5899
			SCMV	JX237862	9571
P2	Papua New Guinea	Spring	SCYLV-CHN	GU190159	5879
		Fall	SCYLV-CHN	GU190159	5879
P3	Australia	Spring	SCSMaV	NC_003870	8146
		Fall	SCSMaV	NC_003870	8146
P4	Australia	Spring	FDV	NC_007159	4532
		Fall	FDV	NC_007159	4532
P5	South Africa	Spring	SCSEV	NC_001868	2706
			SCWSV	NC_023989	2830
		Fall	SCSEV	NC_001868	2706
			SCWSV	NC_023989	2830
P6	USA	Spring	SCYLV-CHN	GU190159	5879
		Fall	SCYLV-CHN	GU190159	5879
P7	Pakistan	Spring	SCSMV	NC_014037	9782
		Fall	SCSMV	NC_014037	9782
P8	Guatemala	Spring	SCYLV-BRA	AF157029	5899
		Fall	SCYLV-BRA	AF157029	5899
P9	Guatemala	Spring	SCYLV-BRA	AF157029	5899
		Fall	SCYLV-BRA	AF157029	5899

^1^ Abbreviations: sugarcane yellow leaf virus-Brazil (SCYLV-BRA); sugarcane mosaic virus (SCMV); sugarcane yellow leaf virus-China (SCYLV-CHN); sugarcane striate mosaic associated virus (SCSMaV); fiji disease virus (FDV); sugarcane streak Egypt virus (SCSEV); sugarcane white streak virus (SCWSV); sugarcane streak mosaic virus (SCSMV).

**Table 3:** Summary of sequencing and mapping statistics from HTS data generated for the nine sugarcane samples carrying various viruses.

Sample	Season	Total Number Reads ^1^	Virus Identified ^2^	No. Viral Reads ^3^	RPKM ^4^	% Viral Reads ^5^	% Viral Genome Coverage	% Identity ^6^
P1	Spring	39,941,799	SCYLV-BRA	348,348	1478	0.87	99	99
			SCMV	1,722,495	4506	4.31	100	96
P1	Fall	34,084,801	SCYLV-BRA	79,964	398	0.23	99	99
			SCMV	837,377	2567	2.46	100	96
P2	Spring	27,907,075	SCYLV-CHN	275,879	1682	0.99	100	99
P2	Fall	25,794,227	SCYLV-CHN	41,048	271	0.16	99	99
P3	Spring	26,379,182	SCSMaV	251,296	1169	0.95	100	98
P3	Fall	42,730,577	SCSMaV	438,761	1261	1.03	100	98
P4	Spring	24,572,155	FDV	217,508	1953	0.89	100	99
P4	Fall	40,623,810	FDV	15,436	84	0.04	100	99
P5	Spring	24,479,398	SCSEV	6414	97	0.03	100	98
			SCWSV	1201	17	4.91 × 10^−3^	94	92
P5	Fall	25,615,770	SCSEV	593	9	2.31 × 10^−3^	100	98
			SCWSV	337	5	1.30 × 10^−3^	79	89
P6	Spring	24,923,563	SCYLV-CHN	240,579	1642	0.97	100	99
P6	Fall	25,630,762	SCYLV-CHN	69,202	459	0.27	99	99
P7	Spring	27,901,105	SCSMV	1,497,825	5488	5.37	100	99
P7	Fall	39,850,078	SCSMV	838,923	2152	2.11	100	99
P8	Spring	32,773,246	SCYLV-BRA	211,597	1094	0.65	99	99
P8	Fall	36,321,988	SCYLV-BRA	58,126	271	0.16	100	98
P9	Spring	31,413,886	SCYLV-BRA	289,497	1562	0.92	100	99
P9	Fall	39,661,498	SCYLV-BRA	21,790	93	0.05	99	99

^1^ Total number of reads obtained per sample; ^2^ Abbreviations: sugarcane yellow leaf virus-Brazil (SCYLV-BRA); sugarcane mosaic virus (SCMV); sugarcane yellow leaf virus-China (SCYLV-CHN); sugarcane striate mosaic associated virus (SCSMaV); fiji disease virus (FDV); sugarcane streak Egypt virus (SCSEV); sugarcane white streak virus (SCWSV); sugarcane streak mosaic virus (SCSMV); ^3^ Total number of unique reads mapped to viral reference sequences; ^4^ RPKM: Reads per kilobase of transcript per million mapped reads; ^5^ Percentage of viral reads from total number of reads obtained per sample; ^6^ Percentage identity between de novo-assembled viral genome and the best-hit viral genome.

## Data Availability

The raw reads from this project are deposited in NCBI SRA under the BioProject ID: PRJNA747280.

## Supplementary Materials

The following are available online at https://www.mdpi.com/article/10.3390/v13081627/s1, Table S1, Summary of read sub-sampling analysis for viral pathogen detection; Table S2, qPCR assays used to validate HTS results; and Table S3, RT-PCR and RT-PCR validation of HTS results.

## References

[1] Oerke E.C. (2006). Crop losses to pests. J. Agric. Sci..

[2] Savary S., Willocquet L., Pethybridge S.J., Esker P., McRoberts N., Nelson A. (2019). The global burden of pathogens and pests on major food crops. Nat. Ecol. Evol..

[3] Sastry K.S., Zitter T.A. (2014). Management of Virus and Viroid Disease of Crops in the Tropics. Plant Virus and Viroid Diseases in the Tropics.

[4] Rybicki E.P. (2015). A Top Ten list for economically important plant viruses. Arch. Virol..

[5] Byrne P.F., Volk G.M., Gardner C., Gore M.A., Simon P.W., Smith S. (2018). Sustaining the future of plant breeding: The critical role of the USDA-ARS national plant germplasm system. Crop Sci..

[6] Savary S., Teng P.S., Willocquet L., Nutter F.W. (2006). Quantification and modeling of crop losses: A review of purposes. Annu. Rev. Phytopathol..

[7] Levy L., Damsteegt V., Welliver R. (2000). First Report of Plum pox virus (Sharka Disease) in Prunus persica in the United States. Plant Dis..

[8] Cambra M., Capote N., Myrta A., Llácer G. (2006). Plum pox virus and the estimated costs associated with sharka disease. EPPO Bull..

[9] Barba M., Hadidi A., Candresse T., Cambra M., Hadidi A., Barba M., Candress T., Jelkmann W. (2011). Plum pox virus. Virus and Virus-Like Diseases of Pome and Stone Fruits.

[10] USDA Declares United States Free from Plum Pox Virus. https://www.usda.gov/media/press-releases/2019/10/17/usda-declares-united-states-free-plum-pox-virus.

[11] Carvajal-Yepes M., Cardwell K., Nelson A., Garrett K.A., Giovani B., Saunders D.G.O., Kamoun S., Legg J.P., Verdier V., Lessel J. (2019). A global surveillance system for crop diseases. Science.

[12] Strange R.N., Scott P.R. (2005). Plant disease: A threat to global food security. Annu. Rev. Phytopathol..

[13] Animal and Plant Health Inspection Service, US. Department of Agriculture Plant Quarantine Programs Managed by APHIS-PPQ. https://www.aphis.usda.gov/aphis/ourfocus/planthealth/import-information/permits/plants-and-plant-products-permits/prohibited/Importation-of-Plant-Parts-for-Propagation/.

[14] Grof C.P.L., Campbell J.A. (2001). Sugarcane sucrose metabolism: Scope for molecular manipulation. Aust. J. Plant Physiol..

[15] Dal-Bianco M., Carneiro M.S., Hotta C.T., Chapola R.G., Hoffmann H.P., Garcia A.A.F., Souza G.M. (2012). Sugarcane improvement: How far can we go?. Curr. Opin. Biotechnol..

[16] Ling H., Huang N., Wu Q., Su Y., Peng Q., Ahmed W., Gao S., Su W., Que Y., Xu L. (2018). Transcriptional Insights into the Sugarcane-Sorghum mosaic virus Interaction. Trop. Plant Biol..

[17] Viswanathan R., Parameswari B., Nithya K. (2018). Molecular Characterization of Sugarcane Viruses and Their Diagnostics. Crop Improvement through Microbial Biotechnology.

[18] Olmos A., Boonham N., Candresse T., Gentit P., Giovani B., Kutnjak D., Liefting L., Maree H.J., Minafra A., Moreira A. (2018). High-throughput sequencing technologies for plant pest diagnosis: Challenges and opportunities. EPPO Bull..

[19] Villamor D.E.V., Ho T., Al Rwahnih M., Martin R.R., Tzanetakis I.E. (2019). High throughput sequencing for plant virus detection and discovery. Phytopathology.

[20] Al Rwahnih M., Daubert S., Golino D., Rowhani A. (2009). Deep sequencing analysis of RNAs from a grapevine showing Syrah decline symptoms reveals a multiple virus infection that includes a novel virus. Virology.

[21] Candresse T., Filloux D., Muhire B., Julian C., Galzi S., Fort G., Bernardo P., Daugrois J.H., Fernandez E., Martin D.P. (2014). Appearances can be deceptive: Revealing a hidden viral infection with deep sequencing in a plant quarantine context. PLoS ONE.

[22] Chiumenti M., Torchetti E.M., Di Serio F., Minafra A. (2014). Identification and characterization of a viroid resembling apple dimple fruit viroid in fig (*Ficus carica* L.) by next generation sequencing of small RNAs. Virus Res..

[23] Bag S., Al Rwahnih M., Li A., Gonzalez A., Rowhani A., Uyemoto J.K., Sudarshana M.R. (2015). Detection of a new luteovirus in imported nectarine trees: A case study to propose adoption of metagenomics in post-entry quarantine. Phytopathology.

[24] Katsiani A., Maliogka V.I., Katis N., Svanella-Dumas L., Olmos A., Ruiz-García A.B., Marais A., Faure C., Theil S., Lotos L. (2018). High-throughput sequencing reveals further diversity of little cherry virus 1 with implications for diagnostics. Viruses.

[25] Bejerman N., Debat H., Dietzgen R.G. (2020). The Plant Negative-Sense RNA Virosphere: Virus Discovery through New Eyes. Front. Microbiol..

[26] Beris D., Ioanna M., Vassilakos N., Theologidis I., Rampou A., Kektsidou O., Massart S., Varveri C. (2021). Association of citrus virus A to citrus impietratura disease symptoms. Phytopathology.

[27] Adams I., Fox A., Wang A., Zhou X. (2016). Diagnosis of Plant Viruses Using Nest-Generation Sequencing and Mategenomic Analysis. Current Research Topics in Plant Virology.

[28] Al Rwahnih M., Daubert S., Golino D., Islas C., Rowhani A. (2015). Comparison of next-generation sequencing versus biological indexing for the optimal detection of viral pathogens in grapevine. Phytopathology.

[29] Bester R., Cook G., Breytenbach J.H.J., Steyn C., De Bruyn R., Maree H.J. (2021). Towards the validation of high-throughput sequencing (HTS) for routine plant virus diagnostics: Measurement of variation linked to HTS detection of citrus viruses and viroids. Virol. J..

[30] Maree H.J., Fox A., Al Rwahnih M., Boonham N., Candresse T. (2018). Application of hts for routine plant virus diagnostics: State of the art and challenges. Front. Plant Sci..

[31] Kinoti W.M., Nancarrow N., Dann A., Rodoni B.C., Constable F.E. (2020). Updating the quarantine status of prunus infecting viruses in Australia. Viruses.

[32] Rott M., Xiang Y., Boyes I., Belton M., Saeed H., Kesanakurti P., Hayes S., Lawrence T., Birch C., Bhagwat B. (2017). Application of next generation sequencing for diagnostic testing of tree fruit viruses and viroids. Plant Dis..

[33] Honjo M.N., Emura N., Kawagoe T., Sugisaka J., Kamitani M., Nagano A.J., Kudoh H. (2020). Seasonality of interactions between a plant virus and its host during persistent infection in a natural environment. ISME J..

[34] Roden L.C., Ingle R.A. (2009). Lights, rhythms, infection: The role of light and the circadian clock in determining the outcome of plant-pathogen interactions. Plant Cell.

[35] Szittya G., Silhavy D., Molnár A., Havelda Z., Lovas Á., Lakatos L., Bánfalvi Z., Burgyán J. (2003). Low temperature inhibits RNA silencing-mediated defence by the control of siRNA generation. EMBO J..

[36] Chellappan P., Vanitharani R., Ogbe F., Fauquet C.M. (2005). Effect of temperature on geminivirus-induced RNA silencing in plants. Plant Physiol..

[37] Szittya G., Burgyán J., Cullen B.R. (2013). RNA Interference-Mediated Intrinsic Antiviral Immunity in Plants. Intrinsic Immunity.

[38] Jones R.W., Jackson A.O., Morris T.J. (1990). Defective-interfering RNAs and elevated temperatures inhibit replication of tomato bushy stunt virus in inoculated protoplasts. Virology.

[39] Ohsato S., Miyanishi M., Shirako Y. (2003). The optimal temperature for RNA replication in cells infected by Soil-borne wheat mosaic virus is 17 °C. J. Gen. Virol..

[40] Chung B.N., Choi K.S., Ahn J.J., Joa J.H., Do K.S., Park K.S. (2015). Effects of temperature on systemic infection and symptom expression of turnip mosaic virus in chinese cabbage (*Brassica campestris*). Plant Pathol. J..

[41] Chung B.N., Canto T., Tenllado F., Choi K.S., Joa J.H., Ahn J.J., Kim C.H., Do K.S. (2016). The effects of high temperature on infection by Potato virus Y, potato virus A, and Potato leafroll virus. Plant Pathol. J..

[42] Kumar A., Kankainen M., Parsons A., Kallioniemi O., Mattila P., Heckman C.A. (2017). The impact of RNA sequence library construction protocols on transcriptomic profiling of leukemia. BMC Genom..

[43] Mortazavi A., Williams B.A., McCue K., Schaeffer L., Wold B. (2008). Mapping and quantifying mammalian transcriptomes by RNA-Seq. Nat. Methods.

[44] Bal A., Pichon M., Picard C., Casalegno J.S., Valette M., Schuffenecker I., Billard L., Vallet S., Vilchez G., Cheynet V. (2018). Quality control implementation for universal characterization of DNA and RNA viruses in clinical respiratory samples using single metagenomic next-generation sequencing workflow. bioRxiv.

[45] Hily J.M., Candresse T., Garcia S., Vigne E., Tannière M., Komar V., Barnabé G., Alliaume A., Gilg S., Hommay G. (2018). High-throughput sequencing and the viromic study of grapevine leaves: From the detection of grapevine-infecting viruses to the description of a new environmental Tymovirales member. Front. Microbiol..

[46] Vigne E., Garcia S., Komar V., Lemaire O., Hily J.M. (2018). Comparison of serological and molecular methods with high-throughput sequencing for the detection and quantification of grapevine fanleaf virus in vineyard samples. Front. Microbiol..

[47] Livak K.J., Schmittgen T.D. (2001). Analysis of relative gene expression data using real-time quantitative PCR and the 2-ΔΔCT method. Methods.

[48] Visser M., Bester R., Burger J.T., Maree H.J. (2016). Next-generation sequencing for virus detection: Covering all the bases. Virol. J..

[49] Ma Y., Marais A., Lefebvre M., Theil S., Svanella-Dumas L., Faure C., Candresse T. (2019). Phytovirome Analysis of Wild Plant Populations: Comparison of Double-Stranded RNA and Virion-Associated Nucleic Acid Metagenomic Approaches. J. Virol..

[50] Pecman A., Kutnjak D., Gutiérrez-Aguirre I., Adams I., Fox A., Boonham N., Ravnikar M. (2017). Next generation sequencing for detection and discovery of plant viruses and viroids: Comparison of two approaches. Front. Microbiol..

[51] Massart S., Chiumenti M., De Jonghe K., Glover R., Haegeman A., Koloniuk I., Komínek P., Kreuze J., Kutnjak D., Lotos L. (2019). Virus detection by high-throughput sequencing of small RNAs: Large-scale performance testing of sequence analysis strategies. Phytopathology.

[52] Maliogka V.I., Minafra A., Saldarelli P., Ruiz-García A.B., Glasa M., Katis N., Olmos A. (2018). Recent advances on detection and characterization of fruit tree viruses using high-throughput sequencing technologies. Viruses.

[53] Süss J., Schrader C., Abel U., Voigt W.P., Schosser R. (1999). Annual and seasonal variation of tick-borne encephalitis virus (TBEV) prevalence in ticks in selected hot spot areas in Germany using a nRT-PCR: Results from 1997 and 1998. Zent. Bakteriol..

[54] Withyachumnarnkul B., Boonsaeng V., Chomsoong R., Flegel T.W., Muangsin S., Nash G.L. (2003). Seasonal variation in white spot syndrome virus-positive samples in broodstock and post-larvae of Penaeus monodon in Thailand. Dis. Aquat. Organ..

[55] Van Dijk J.G.B., Hoye B.J., Verhagen J.H., Nolet B.A., Fouchier R.A.M., Klaassen M. (2014). Juveniles and migrants as drivers for seasonal epizootics of avian influenza virus. J. Anim. Ecol..

[56] Agha S.B., Tchouassi D.P., Bastos A.D.S., Sang R. (2017). Dengue and yellow fever virus vectors: Seasonal abundance, diversity and resting preferences in three Kenyan cities. Parasites Vectors.

[57] Llamas-Llamas M.E., Zavaleta-Mejia E., Gonzalez-Hernandez V.A., Cervantes-Diaz L., Santizo-Rincon J.A., Ochoa-Martinez D.L. (1998). Effect of temperature on symptom expression and accumulation of tomato spotted wilt virus in different host species. Plant Pathol..

[58] Hull R., Hull R. (2013). Replication of Plant Viruses. Plant Virology.

[59] Li Z., Nagy P.D. (2011). Diverse roles of host RNA binding proteins in RNA virus replication. RNA Biol..

[60] Fondong V.N. (2013). Geminivirus protein structure and function. Mol. Plant Pathol..

