# Supplementary material for: HTS-Based Diagnostics of Sugarcane Viruses: Seasonal Variation and Its Implications for Accurate Detection
Source: Viruses. 2021 Aug 17;13(8):1627. doi: 10.3390/v13081627 (PMC8402784; doi:10.3390/v13081627)
Supplement: Supplementary file 1 [file viruses-13-01627-s001.zip › viruses-1323501-supplementary.pdf]

Table S1: Summary of read sub-sampling analysis for viral pathogen detection.

Sample	Season*	No. of reads sampled[Π]	Virus identified[Ψ]	Accession of the top hit[δ]	Viral genome size (bp)[Ω]	Average no. of mapped reads[ϕ]	SE of mapped reads	% genome coverage[β]
P8	Fall	500,000	SCYLV-BRA	AF157029	5899	762	18	98
		1,000,000				1,624	37	99
		5,000,000				8,086	51	99
		10,000,000				16,076	55	99
		20,000,000				31,993	22	99
P8	Spring	500,000	SCYLV-BRA	AF157029	5899	3,214	30	99
		1,000,000				6,379	25	99
		5,000,000				32,318	100	99
		10,000,000				64,668	67	99
		20,000,000				129,088	148	99
P9	Fall	500,000	SCYLV-BRA	AF157029	5899	275	8	92
		1,000,000				534	9	98
		5,000,000				2,796	28	99
		10,000,000				5,456	9	99
		20,000,000				11,066	48	99
P9	Spring	500,000	SCYLV-BRA	AF157029	5899	4,605	7	99
		1,000,000				9,153	90	99
		5,000,000				45,975	64	99
		10,000,000				92,096	168	100
		20,000,000				184,249	198	100
P4	Fall	500,000	FDV	NC_007159	4532	189	9	92
		1,000,000				376	16	98
		5,000,000				1,861	34	100
		10,000,000				3,762	18	100
		20,000,000				7,618	12	100
P4	Spring	500,000	FDV	NC_007159	4532	4,408	18	100
		1,000,000				8,866	49	100
		5,000,000				44,373	8	100
		10,000,000				88,408	68	100
		20,000,000				176,904	66	100
P3	Fall	500,000	SCSMaV	NC_003870	8146	5,141	50	100
		1,000,000				10,337	20	100
		5,000,000				51,432	177	100
		10,000,000				102,587	191	100
		20,000,000				205,661	231	100
P3	Spring	500,000	SCSMaV	NC_003870	8146	4,813	41	100
		1,000,000				9,456	84	100

		5,000,000				47,742	75	100
		10,000,000				95,313	250	100
		20,000,000				190,695	44	100
P7	Fall	500,000	SCSMV	NC_014037	9782	10,461	22	100
		1,000,000				20,859	101	100
		5,000,000				105,303	124	100
		10,000,000				210,558	77	100
		20,000,000				420,743	100	100
		25,000,000				527,457	924	100
		30,000,000				632,028	116	100
		35,000,000				736,891	333	100
P7	Spring	500,000	SCSMV	NC_014037	9782	26,971	54	100
		1,000,000				53,759	71	100
		5,000,000				267,685	75	100
		10,000,000				536,240	245	100
		20,000,000				1,073,055	479	100
		25,000,000				1,341,120	577	100
P1	Spring	500,000	SCMV	JX237862	9571	21,596	113	100
		1,000,000				43,037	81	100
		5,000,000				215,995	82	100
		10,000,000				431,270	383	100
		20,000,000				862,117	244	100
P1	Fall	500,000	SCMV	JX237862	9571	12,287	11	100
		1,000,000				24,671	87	100
		5,000,000				122,930	294	100
		10,000,000				245,268	140	100
		20,000,000				491,477	155	100
P1	Spring	500,000	SCYLV-BRA	AF157029	5899	4,323	19	99
		1,000,000				8,764	20	99
		5,000,000				43,432	168	99
		10,000,000				86,899	177	99
		20,000,000				174,245	157	99
P1	Fall	500,000	SCYLV-BRA	AF157029	5899	1,147	27	99
		1,000,000				2,378	43	99
		5,000,000				11,835	58	99
		10,000,000				23,470	52	99
		20,000,000				46,844	123	99
P5	Spring	500,000	SCSEV	NC_001868	2706	129	3	84
		1,000,000				256	8	93
		5,000,000				1,296	9	100
		10,000,000				2,615	2	100

		20,000,000				5,254	16	100
		25,000,000				6,402	6	100
P5	Fall	500,000	SCSEV	NC_001868	2706	13	1	30
		1,000,000				22	1	46
		5,000,000				111	7	94
		10,000,000				233	8	100
		20,000,000				469	2	100
		25,000,000				581	2	100
P5	Spring	500,000	SCWSV	NC_023989	2830	24	1	30
		1,000,000				45	5	44
		5,000,000				253	6	72
		10,000,000				503	4	87
		20,000,000				970	9	92
		25,000,000				1,199	1	94
P5	Fall	500,000	SCWSV	NC_023989	2830	6	2	14
		1,000,000				12	0	23
		5,000,000				82	17	53
		10,000,000				144	16	65
		20,000,000				262	2	73
		25,000,000				330	0	79
P6	Spring	500,000	SCYLV-CHN	GU190159	5879	4,768	84	99
		1,000,000				9,630	100	99
		5,000,000				48,193	73	99
		10,000,000				96,912	158	99
		20,000,000				193,051	93	99
P6	Fall	500,000	SCYLV-CHN	GU190159	5879	1,354	18	100
		1,000,000				2,688	22	100
		5,000,000				13,442	38	100
		10,000,000				26,890	21	100
		20,000,000				54,012	88	100
P2	Spring	500,000	SCYLV-CHN	GU190159	5879	4,967	31	99
		1,000,000				9,910	15	99
		5,000,000				49,356	71	100
		10,000,000				98,995	87	100
		20,000,000				197,759	187	100
P2	Fall	500,000	SCYLV-CHN	GU190159	5879	804	4	100
		1,000,000				1,561	25	100
		5,000,000				7,967	12	100
		10,000,000				15,921	56	100
		20,000,000				32,108	318	100

Both RNA (names in bold) and DNA viruses were identified and validated using CLC Genomics Workbench.

Abbreviations: *Sugarcane yellow leaf virus*-Brazil (SCYLV-BRA); *Sugarcane mosaic virus* (SCMV); *Sugarcane yellow leaf virus*-China (SCYLV-CHN); *Sugarcane striate mosaic associated virus* (SCSMaV); *Fiji disease virus* (FDV); *Sugarcane streak Egypt virus* (SCSEV); *Sugarcane white streak virus* (SCWSV); *Sugarcane streak mosaic virus* (SCSMV); standard error (SE).

[*]Season of sampling and RNA extraction.

[Π]Sequence reads were randomly sampled using the 'Sample Reads' feature under NGS Core Tools in CLC Genomics Workbench.

[Ψ]Viral pathogens were identified by mapping assembled contigs to virus database using BLAST program implemented in CLC Genomics Workbench.

[δ]GenBank accession of the top viral hit identified by BLAST analysis.

[Ω]Genome size of the best hit accession from GenBank.

[ϕ]Average number of sequencing reads mapped to best hit viral genome obtained from BLAST analysis.

[β]Average genome coverage of sequence reads mapped to best hit viral genome

Table S2. qPCR assays used to validate HTS results

Target	Forward Primer (5'- 3')	Probe (5'- 3')[1,2]	Reverse Primer (5'- 3')
**SCYLV**	GGCTCCAAGATTACTAGCTTCC	CGTCAAGAGGAACGCCAAGAAAGTCT	CTCGAGTCTCCATTCCCTTTG
**SCSMV**	CGTAGCGGGAAACCCATAATAC	CACACAATGCAGTGAAGGCAGGAT	GATTTCTGCTGGTGAGAGGATG
**SCSEV**	GCGGGCGATTTACTTCCATAA	CCTTGGTGTGAAGACGGAGTGGAA	GGTACAACGCACCCTTCTTAAT
**SCWSV**	AAAGACGAGGAGCGGAAAC	TCAAGCTGGTGTTCGCTGTAACCT	CACATCCCACGGGATACATATT
**FDV**	TCAAGAATGAAGAACAGTCTGA	CGCGAAGAATTGTAGTTGAATTGGT	GGAGTGTATTCAATTTCGTCTCT
**SCSMaV**	GAACCAGAAGGTGGGTTACTT	TGAGAGCTGCAAACATGGCTAGGA	CCTCCCTTTCTCTCACTTCTTT
**SCMV**	CAGCGAAATCTCACCGACTAT	ACGAAATGACTTCAAGGACACCAGCT	CTGCATGTGGGCTTCCTTA
**Nad5[3]**	GATGCTTCTTGGGGCTTCTTKTT	CATAAGTAGCTTGGTCCATCTTTATTCCAT	ACATAAATCGAGGGCTATGCGGATC

[1] For viruses, 5' fluorescent dye and 3' quencher is FAM and IBFQ, respectively; internal quencher ZEN is added.

[2] For Nad5, 5' fluorescent dye and 3' quencher is VIC and BHQ-1, respectively.

[3] Designed by USDA APHIS S&T Beltsville Laboratory.

Table S3. RT-PCR and RT-PCR validation of HTS results

	RT-PCR	qRT-PCR		
		**Ct (CP)**	**Ct (Nad5)**	**Seasonal Change (Fall/Spring)**
**RNA Viruses/Sample**				
SCYLV-Sp/P1	Positive	17.49	19.70	18.04
SCYLV-Fa/P1	Positive	20.30	18.33	
SCMV-Sp/P1	Positive	14.10	17.87	2.32
SCMV-Fa/P1	Positive	15.06	17.62	
SCYLV-Sp/P2	Positive	19.99	20.35	2.58
SCYLV-Fa/P2	Positive	18.77	17.77	
SCSMaV-Sp/P3	Positive	17.93	19.98	0.92
SCSMaV-Fa/P3	Positive	17.20	19.37	
FDV-Sp/P4	Positive	15.73	20.32	77.90
FDV-Fa/P4	Positive	20.51	18.81	
SCYLV-Sp/P6	Positive	17.32	20.96	4.56
SCYLV-Fa/P6	Positive	19.67	21.12	
SCSMV-Sp/P7	Positive	15.40	19.61	8.31
SCSMV-Fa/P7	Positive	17.40	18.55	
SCYLV-Sp/P8	Positive	17.56	20.41	3.00
SCYLV-Fa/P8	Positive	19.38	20.65	
SCYLV-Sp/P9	Positive	18.46	20.42	36.92
SCYLV-Fa/P9	Positive	23.18	19.94	
**DNA Viruses/Sample**				
SCSEV-Sp/P5	Positive	14.83	21.16	7.41
SCSEV-Fa/P5	Positive	15.63	19.05	
SCWSV-Sp/P5	Positive	22.05	21.44	1.20
SCWSV-Fa/P5	Positive	20.81	19.95	
